# 3,9-Di-*tert*-butyl-2,4,8,10-tetra­oxaspiro­[5.5]undeca­ne

**DOI:** 10.1107/S1600536810049524

**Published:** 2010-11-30

**Authors:** Zhengyi Li, Liang Chen, Qiuzheng Tang, Xiaoqiang Sun

**Affiliations:** a Key Laboratory of Fine Chemical Engineering, Changzhou University, Changzhou 213164, Jiangsu, People’s Republic of China

## Abstract

The title compound, C_15_H_28_O_4_, was prepared by the condensation of pivalaldehyde with penta­erythritol. In the crystal, the two halves of the mol­ecule are related by a crystallographic twofold rotation axis passing through the central spiro-C atom. The two non-planar six-membered heterocycles both adopt chair conformations with the two *tert*-butyl groups both located in the equatorial positions.

## Related literature

For general background to spiranes, see: Cismaş *et al.* (2005 ▶); Mihiş *et al.* (2008 ▶); Sun *et al.* (2010 ▶).
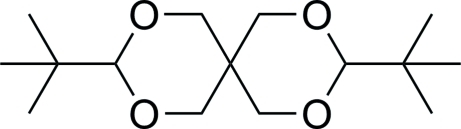


## Experimental

### 

#### Crystal data

C_15_H_28_O_4_
*M* *_r_* = 272.37
Monoclinic, 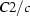

*a* = 26.726 (4) Å
*b* = 5.7894 (8) Å
*c* = 11.2635 (15) Å
β = 113.846 (4)°
*V* = 1594.0 (4) Å^3^
*Z* = 4
Mo *K*α radiation
μ = 0.08 mm^−1^
*T* = 295 K
0.35 × 0.32 × 0.15 mm

#### Data collection

Bruker APEXII CCD diffractometer
Absorption correction: multi-scan (*SADABS*; Bruker, 2000 ▶) *T*_min_ = 0.972, *T*_max_ = 0.988
4400 measured reflections
1513 independent reflections
1347 reflections with *I* > 2σ(*I*)
*R*_int_ = 0.019

#### Refinement

*R*[*F* ^2^ > 2σ(*F* ^2^)] = 0.042
*wR*(*F* ^2^) = 0.145
*S* = 1.03
1513 reflections
90 parameters
H-atom parameters constrained
Δρ_max_ = 0.21 e Å^−3^
Δρ_min_ = −0.25 e Å^−3^

### 

Data collection: *APEX2* (Bruker, 2000 ▶); cell refinement: *SAINT* (Bruker, 2000 ▶); data reduction: *SAINT*; program(s) used to solve structure: *SHELXS97* (Sheldrick, 2008 ▶); program(s) used to refine structure: *SHELXL97* (Sheldrick, 2008 ▶); molecular graphics: *SHELXTL* (Sheldrick, 2008 ▶); software used to prepare material for publication: *SHELXL97*.

## Supplementary Material

Crystal structure: contains datablocks I, global. DOI: 10.1107/S1600536810049524/si2313sup1.cif

Structure factors: contains datablocks I. DOI: 10.1107/S1600536810049524/si2313Isup2.hkl

Additional supplementary materials:  crystallographic information; 3D view; checkCIF report

## Figures and Tables

**Fig. 1.**
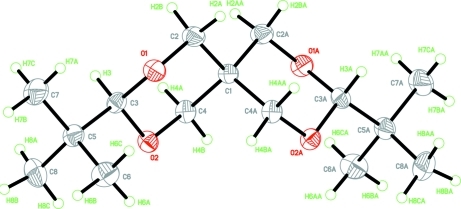
The molecular structure of the title compound. Displacement ellipsoids are drawn at the 30% probability level. [symmetry code: -x, y, -z + 1/2].

## supplementary crystallographic information

### Comment

Owing to the characteristic axial and helical chirality, the stereochemistry of spiranes with six-membered rings has been extensively studied (Cismaş *et al.*, 2005). In the past three decades, most of these investigations were carried out with spiranes containing 1,3-dioxane units (Mihiş *et al.*, 2008; Sun *et al.*, 2010). We herein present the structure of 3,9-di(*tert*-butyl)-2,4,8,10-tetraoxaspiro[5.5]undecane (Fig. 1).

In the title compound, a 2-fold rotation axis passes through the central spiro-C atom (C1). The two non-planar sixmembered heterocycle [(O1/O2/C1–C4) and (O1A/O2A/C1/C2A–C4A)] both adopt chair conformations. The two *tert*-butyl groups locate at the equatorial position of C3 and C3A in the two six-member *O*-heterocycles, respectively, which give the title molecule with minimum conformational energy.

### Experimental

To a solution of pivaldehyde (7.3 mmol,0.63 g) and pentaerythritol (4 mmol, 0.54 g) in toluene (30 ml), phosphotungstic acid (30 mg) was added as catalyst. The mixtures were refluxed for 6 h to complete the reaction. After reaction, the solvent was evaporated under vacuum and the resulting solid was washed with 5% sodium bicarbonate (20 ml) and 50% ethanol (20 ml). The pure product recrystallized from ethanol to afford a white solid (65% yield, m.p. 451–452 K). Single crystals suitable for X-ray diffraction were also obtained by evaporation of an ethanol solution.

### Refinement

All the H atoms were placed in geometrically idealized positions and constrained to ride on their parent atoms, with C—H distances of 0.96–0.98 Å, and with *U*_iso_(H) = 1.2*U*_eq_(C) or 1.5*U*_eq_(C_methyl_).

### Crystal data

**Table:**

C_15_H_28_O_4_	*F*(000) = 600
*M**_r_* = 272.37	*D*_x_ = 1.135 Mg m^−^^3^
Monoclinic, *C*2/*c*	Melting point = 451–452 K
Hall symbol: -C 2yc	Mo *K*α radiation, λ = 0.71073 Å
*a* = 26.726 (4) Å	Cell parameters from 3133 reflections
*b* = 5.7894 (8) Å	θ = 3.1–25.8°
*c* = 11.2635 (15) Å	µ = 0.08 mm^−^^1^
β = 113.846 (4)°	*T* = 295 K
*V* = 1594.0 (4) Å^3^	Block, colorless
*Z* = 4	0.35 × 0.32 × 0.15 mm

### Data collection

**Table:**

Bruker APEXII CCD diffractometer	1513 independent reflections
Radiation source: fine-focus sealed tube	1347 reflections with *I* > 2σ(*I*)
graphite	*R*_int_ = 0.019
φ and ω scans	θ_max_ = 25.8°, θ_min_ = 3.3°
Absorption correction: multi-scan (*SADABS*; Bruker, 2000)	*h* = −26→32
*T*_min_ = 0.972, *T*_max_ = 0.988	*k* = −6→7
4400 measured reflections	*l* = −13→13

### Refinement

**Table:**

Refinement on *F*^2^	Primary atom site location: structure-invariant direct methods
Least-squares matrix: full	Secondary atom site location: difference Fourier map
*R*[*F*^2^ > 2σ(*F*^2^)] = 0.042	Hydrogen site location: inferred from neighbouring sites
*wR*(*F*^2^) = 0.145	H-atom parameters constrained
*S* = 1.03	*w* = 1/[σ^2^(*F*_o_^2^) + (0.1*P*)^2^ + 0.4*P*] where *P* = (*F*_o_^2^ + 2*F*_c_^2^)/3
1513 reflections	(Δ/σ)_max_ < 0.001
90 parameters	Δρ_max_ = 0.21 e Å^−^^3^
0 restraints	Δρ_min_ = −0.25 e Å^−^^3^

### Special details

**Table:**

Geometry. All e.s.d.'s (except the e.s.d. in the dihedral angle between two l.s. planes) are estimated using the full covariance matrix. The cell e.s.d.'s are taken into account individually in the estimation of e.s.d.'s in distances, angles and torsion angles; correlations between e.s.d.'s in cell parameters are only used when they are defined by crystal symmetry. An approximate (isotropic) treatment of cell e.s.d.'s is used for estimating e.s.d.'s involving l.s. planes.
Refinement. Refinement of *F*^2^ against ALL reflections. The weighted *R*-factor *wR* and goodness of fit *S* are based on *F*^2^, conventional *R*-factors *R* are based on *F*, with *F* set to zero for negative *F*^2^. The threshold expression of *F*^2^ > σ(*F*^2^) is used only for calculating *R*-factors(gt) *etc*. and is not relevant to the choice of reflections for refinement. *R*-factors based on *F*^2^ are statistically about twice as large as those based on *F*, and *R*- factors based on ALL data will be even larger.

### Fractional atomic coordinates and isotropic or equivalent isotropic displacement parameters (Å^2^)

**Table:**

	*x*	*y*	*z*	*U*_iso_*/*U*_eq_	
C1	0.0000	0.5543 (3)	0.2500	0.0352 (4)	
C2	0.03372 (5)	0.4010 (2)	0.36479 (11)	0.0457 (4)	
H2A	0.0094	0.3201	0.3953	0.055*	
H2B	0.0531	0.2865	0.3366	0.055*	
C3	0.10728 (4)	0.66075 (19)	0.42765 (10)	0.0374 (3)	
H3	0.1261	0.5544	0.3918	0.045*	
C4	0.03957 (4)	0.7056 (2)	0.21734 (10)	0.0387 (3)	
H4A	0.0598	0.6105	0.1811	0.046*	
H4B	0.0191	0.8192	0.1527	0.046*	
C5	0.14972 (5)	0.7871 (2)	0.54331 (11)	0.0433 (3)	
C6	0.12174 (6)	0.9403 (3)	0.60883 (13)	0.0582 (4)	
H6A	0.0987	1.0505	0.5473	0.087*	
H6B	0.1490	1.0204	0.6805	0.087*	
H6C	0.1000	0.8462	0.6398	0.087*	
C7	0.18565 (6)	0.6080 (3)	0.64011 (15)	0.0683 (5)	
H7A	0.1634	0.5132	0.6692	0.102*	
H7B	0.2128	0.6855	0.7131	0.102*	
H7C	0.2034	0.5130	0.5988	0.102*	
C8	0.18477 (6)	0.9364 (3)	0.49431 (15)	0.0643 (4)	
H8A	0.1981	0.8436	0.4427	0.096*	
H8B	0.2152	0.9985	0.5670	0.096*	
H8C	0.1630	1.0607	0.4426	0.096*	
O1	0.07228 (3)	0.53230 (14)	0.46900 (7)	0.0424 (3)	
O2	0.07695 (3)	0.82062 (13)	0.33090 (7)	0.0382 (3)	

### Atomic displacement parameters (Å^2^)

**Table:**

	*U*^11^	*U*^22^	*U*^33^	*U*^12^	*U*^13^	*U*^23^
C1	0.0386 (8)	0.0345 (8)	0.0284 (8)	0.000	0.0094 (6)	0.000
C2	0.0484 (7)	0.0370 (6)	0.0406 (7)	−0.0019 (5)	0.0065 (6)	0.0047 (5)
C3	0.0360 (6)	0.0408 (6)	0.0326 (6)	0.0058 (4)	0.0111 (5)	0.0006 (4)
C4	0.0391 (6)	0.0486 (7)	0.0266 (5)	−0.0005 (5)	0.0114 (5)	−0.0003 (4)
C5	0.0410 (6)	0.0457 (7)	0.0337 (6)	0.0034 (5)	0.0052 (5)	−0.0004 (5)
C6	0.0714 (9)	0.0581 (8)	0.0399 (7)	0.0047 (7)	0.0173 (6)	−0.0101 (6)
C7	0.0557 (8)	0.0646 (9)	0.0557 (8)	0.0097 (7)	−0.0074 (7)	0.0053 (7)
C8	0.0473 (7)	0.0774 (11)	0.0579 (9)	−0.0140 (7)	0.0107 (6)	−0.0029 (7)
O1	0.0457 (5)	0.0433 (5)	0.0310 (5)	−0.0019 (3)	0.0081 (4)	0.0060 (3)
O2	0.0382 (5)	0.0424 (5)	0.0296 (5)	−0.0031 (3)	0.0090 (4)	0.0028 (3)

### Geometric parameters (Å, °)

**Table:**

C1—C2^i^	1.5261 (14)	C4—H4B	0.9700
C1—C2	1.5261 (14)	C5—C6	1.5288 (18)
C1—C4^i^	1.5281 (14)	C5—C7	1.5293 (17)
C1—C4	1.5282 (14)	C5—C8	1.5322 (19)
C2—O1	1.4281 (14)	C6—H6A	0.9600
C2—H2A	0.9700	C6—H6B	0.9600
C2—H2B	0.9700	C6—H6C	0.9600
C3—O2	1.4096 (12)	C7—H7A	0.9600
C3—O1	1.4132 (13)	C7—H7B	0.9600
C3—C5	1.5244 (15)	C7—H7C	0.9600
C3—H3	0.9800	C8—H8A	0.9600
C4—O2	1.4299 (13)	C8—H8B	0.9600
C4—H4A	0.9700	C8—H8C	0.9600
			
C2^i^—C1—C2	108.90 (12)	C3—C5—C7	108.66 (10)
C2^i^—C1—C4^i^	107.94 (6)	C6—C5—C7	109.75 (11)
C2—C1—C4^i^	111.00 (7)	C3—C5—C8	108.36 (10)
C2^i^—C1—C4	111.00 (7)	C6—C5—C8	109.63 (11)
C2—C1—C4	107.94 (6)	C7—C5—C8	109.89 (11)
C4^i^—C1—C4	110.06 (13)	C5—C6—H6A	109.5
O1—C2—C1	111.69 (9)	C5—C6—H6B	109.5
O1—C2—H2A	109.3	H6A—C6—H6B	109.5
C1—C2—H2A	109.3	C5—C6—H6C	109.5
O1—C2—H2B	109.3	H6A—C6—H6C	109.5
C1—C2—H2B	109.3	H6B—C6—H6C	109.5
H2A—C2—H2B	107.9	C5—C7—H7A	109.5
O2—C3—O1	110.49 (8)	C5—C7—H7B	109.5
O2—C3—C5	110.02 (9)	H7A—C7—H7B	109.5
O1—C3—C5	109.50 (8)	C5—C7—H7C	109.5
O2—C3—H3	108.9	H7A—C7—H7C	109.5
O1—C3—H3	108.9	H7B—C7—H7C	109.5
C5—C3—H3	108.9	C5—C8—H8A	109.5
O2—C4—C1	110.67 (7)	C5—C8—H8B	109.5
O2—C4—H4A	109.5	H8A—C8—H8B	109.5
C1—C4—H4A	109.5	C5—C8—H8C	109.5
O2—C4—H4B	109.5	H8A—C8—H8C	109.5
C1—C4—H4B	109.5	H8B—C8—H8C	109.5
H4A—C4—H4B	108.1	C3—O1—C2	111.29 (8)
C3—C5—C6	110.53 (10)	C3—O2—C4	111.17 (8)
			
C2^i^—C1—C2—O1	171.24 (12)	O1—C3—C5—C7	64.11 (12)
C4^i^—C1—C2—O1	−70.06 (12)	O2—C3—C5—C8	−54.91 (12)
C4—C1—C2—O1	50.64 (12)	O1—C3—C5—C8	−176.52 (10)
C2^i^—C1—C4—O2	−170.67 (8)	O2—C3—O1—C2	61.88 (11)
C2—C1—C4—O2	−51.39 (12)	C5—C3—O1—C2	−176.78 (9)
C4^i^—C1—C4—O2	69.89 (7)	C1—C2—O1—C3	−56.61 (11)
O2—C3—C5—C6	65.24 (12)	O1—C3—O2—C4	−63.27 (10)
O1—C3—C5—C6	−56.37 (13)	C5—C3—O2—C4	175.71 (8)
O2—C3—C5—C7	−174.28 (10)	C1—C4—O2—C3	58.78 (11)

Symmetry codes: (i) −*x*, *y*, −*z*+1/2.
